# The Role of the Transcriptional Response to DNA Replication Stress

**DOI:** 10.3390/genes8030092

**Published:** 2017-03-02

**Authors:** Anna E. Herlihy, Robertus A.M. de Bruin

**Affiliations:** 1 Medical Research Council Laboratory for Molecular Cell Biology, University College London, London WC1E 6BT, UK; a.herlihy.12@ucl.ac.uk; 2 The UCL Cancer Institute, University College London, London WC1E 6BT, UK

**Keywords:** DNA replication, DNA replication stress, checkpoint response, Chk1, E2F-dependent transcription, E2F6, oncogene-induced replication stress

## Abstract

During DNA replication many factors can result in DNA replication stress. The DNA replication stress checkpoint prevents the accumulation of replication stress-induced DNA damage and the potential ensuing genome instability. A critical role for post-translational modifications, such as phosphorylation, in the replication stress checkpoint response has been well established. However, recent work has revealed an important role for transcription in the cellular response to DNA replication stress. In this review, we will provide an overview of current knowledge of the cellular response to DNA replication stress with a specific focus on the DNA replication stress checkpoint transcriptional response and its role in the prevention of replication stress-induced DNA damage.

## 1. DNA Replication

The genome must be faithfully replicated in each cell cycle. In eukaryotic cells, to ensure timely completion of genome duplication, DNA replication is initiated in S phase from multiple origins throughout the genome. To prevent genome instability, DNA must be replicated once and only once during each cell cycle. Re-replication can result in gene amplification and DNA damage [1] but is prevented by a variety of mechanisms. The control of origins of replication has been reviewed previously in [1]. In short, DNA replication is tightly regulated via two distinct and temporally separated stages. Origins are ”licensed” in G1 phase when Cyclin-Dependent Kinase (CDK) activity is low and replication is initiated (origin firing) from these licensed origins in the subsequent S phase, when CDK activity accumulates [2]. Licensing in G1 phase, when CDK activity is low, defines potential sites of replication initiation and occurs through the loading of the Mcm2–7 helicase by the Origin Recognition Complex (ORC, Orc1-6), Cdc6 and Cdt1, forming the pre-Replicative Complex (pre-RC) [1,3,4,5]. The firing of this Mcm2–7 double hexamer is prevented in G1 by low CDK activity. In each G1 phase many more origins are licensed than are used in the following S phase [6]. This results in dormant origins that are not fired in an unperturbed cell cycle, but are important for the response to DNA replication stress [7,8,9]. Dormant origins are disassembled by passive replication in S phase, preventing their activation and re-replication [10].

Progression into S phase requires high CDK activity, which triggers firing of origins and replication initiation. Components of the pre-RC are phosphorylated by the Dbf4-dependent kinase (DDK or Cdc-Dbf4 complex) and Cyclin/CDKs [11,12]. This allows the recruitment of Cdc45, GINS complex, RECQL4 (Sld2 in yeast) and Mcm10, forming the Cdc45/Mcm2–7/GINS (CMG) complex. CDK phosphorylation of Treslin (Sld3 in yeast) and its subsequent interaction with TopBP1 (Dbp11 in yeast) and the CMG complex activates the CMG complex. This initiates replication bidirectionally, with each Mcm2–7 hexamer forming a replication fork and unwinding DNA outwards from the origin [13]. The replication fork is a structure containing the DNA helicase, DNA polymerases, proliferating cell nuclear antigen (PCNA), checkpoint mediators and other proteins. The process of DNA replication requires the exposure of short stretches of single-stranded DNA (ssDNA) between the helicase and lagging-strand polymerase, which is protected by Replication Protein A (RPA), a ssDNA binding protein [14].

Re-licensing, and therefore potential re-replication, is prevented in S phase by a number of mechanisms. Assembly of new pre-RCs is prevented by phosphorylation of pre-RC components, due to high Cyclin/CDK levels in S phase [15]. In metazoans, Geminin also binds to the pre-RC component Cdt1, further preventing new pre-RC formation [16]. This inhibition is relieved in the following G1 phase by anaphase promoting complex/cyclosome-dependent (APC/C-dependent) degradation of Cyclins and Geminin [7]. Cullin-based E3 ubiquitin ligase activity also targets Cdt1 and Orc1 for degradation to prevent re-licensing and re-replication [17,18].

## 2. DNA Replication Stress

The slowing down or stalling of replication forks and exposure of extended lengths of ssDNA, known as DNA replication stress [19], can generate DNA damage. Stalled replication forks can result in inappropriate intermediate structures, which must be resolved to prevent DNA damage and allow completion of DNA replication [20]. In addition, stalled forks can collapse after prolonged periods of stalling, resulting in the dissociation of the replisome complex from DNA [21]. Collapsed forks cannot reinitiate replication and nearby dormant origins must fire to complete DNA replication. The slow progression of replication forks and the ensuing checkpoint-dependent global inhibition of origin firing increases the time required for genome duplication [22]. The end of S phase must therefore be delayed to ensure that all DNA is replicated before the cell enters mitosis.

DNA replication stress can be induced by oncogene activation or tumour-suppressor inactivation. This oncogene-induced replication stress has been extensively reviewed previously [23,24,25]. Oncogene-induced replication stress has recently been proposed as a hallmark of cancer as a very early event in tumourigenesis [26,27,28,29]. Oncogene-induced replication stress is thought to induce DNA damage, with the DNA damage response (reviewed in [30,31]) acting as an initial barrier to tumourigenesis through oncogene-induced senescence or apoptosis [25,32,33]. Replication stress-induced DNA damage is thought to drive mutations that bypass the DNA damage checkpoint and therefore allow continued tumour progression [25]. As such, oncogene-induced replication stress has a key role in the evolution of cancer [24] and understanding the response to replication stress has important implications in enhancing our knowledge of cancer development.

We will now summarise the key causes of DNA replication stress, with reference to how oncogene-induced replication stress may act through these mechanisms where appropriate.

### 2.1. DNA Characteristics

DNA replication stress can be caused by particular DNA sequences that are inherently difficult to replicate [34]. Repeats (dinucleotide, trinucleotide, inverted or tandem) and other sequences can form secondary DNA structures, such as G-quadruplexes, hairpins and z-DNA, which can block replication fork progression [20,35]. Replication through repeats can also induce slippage and subsequent repeat expansion [36]. Areas of the genome containing low origin density can also be inherently difficult to replicate, due to a lack of dormant origins available to rescue stalled forks. Sites of the genome displaying high rates of replication fork stalling and breakage, even following mild replication stress, are known as Common Fragile Sites (CFSs). CFSs show high levels of DNA double-strand breaks (DSBs) and chromosome rearrangements. In early tumourigenesis, these CFSs are frequently the sites of allelic imbalances [26,27]. Although the exact cause of CFS is under debate, it is likely to be due to some or all of the characteristics described above [37,38].

### 2.2. Obstructions to Replication Fork Progression

Proteins tightly bound to DNA can obstruct replication fork progression, resulting in replication stress. DNA is packaged into chromatin and is therefore tightly associated with histone proteins. Heterochromatic regions show increased levels of DNA damage, suggesting that chromatin state can affect DNA replication [39,40]. Other proteins, such as the pre-RC at dormant origins and the kinetochore at centromeres, must be tightly bound to DNA for their function but this can obstruct replication forks and cause topological and replication stress [41,42]. In the case of Replication Fork Barriers (RFBs), proteins are recruited to DNA to deliberately stall replication forks; these barriers are often unidirectional and prevent collisions between replication forks and transcriptional bubbles, discussed below [43]. Replication fork progression can also be halted by bulky lesions formed by DNA damage. The effects of DNA damage on replication will vary depending on the particular lesion. DNA damage and its effects on replication has been extensively reviewed previously [20,31,44].

### 2.3. Replication and Transcription Collisions

Replication forks and transcriptional bubbles move along the same template and can therefore collide. These collisions can generate topological stress [41], thereby causing a slowing down or stalling of replication forks, i.e., DNA replication stress. Collisions between replication forks and transcriptional bubbles can result in the formation of R-loops [19,45]. R-loops are RNA:DNA hybrids formed between nascent RNA transcripts and one DNA strand, with the other DNA strand excluded as ssDNA. R-loops can hinder replication fork progression, expose vulnerable ssDNA and may result in DSBs following transcription-coupled nucleotide excision repair [46]. Spatial and temporal separation of replication and transcription can reduce collisions, but cannot completely prevent them, especially in long or actively transcribed genes [45]. Replication and transcription collisions are thought to be an important mechanism of oncogene-induced replication stress. Activation of the oncogene Cyclin E increases the rates of replication initiation. This misregulation of the replication programme is thought to result in increased replication and transcription collisions, resulting in replication stress [47]. Overexpression of another oncogene, HRAS^V12^, instead increases transcription levels to increase the frequency of collisions and cause replication stress [48].

### 2.4. Loss of Regulation of DNA Replication

Components essential for DNA replication must be present at sufficient levels to support replication at all forks. Depletion of essential components causes replication forks to stall. Most notably, the levels of the four dinucleotide triphosphates (dNTPs) must be sufficient, their levels are primarily controlled by ribonucleotide reductase (RNR) enzyme activity [49]. Increased replication initiation, for example following Cyclin E activation, depletes pools of dNTPs and causes stress, this can be rescued with the addition of exogenous nucleosides [50].

As well as replication component deregulation, loss of control of DNA replication initiation can also cause replication stress, through either increasing or decreasing the frequency of replication initiation. Oncogene activation can drive S phase entry, thereby shortening G1 phase and reducing the number of origins licensed, as seen for Cyclin E [51,52]. Fewer licensed origins or a reduction in limiting firing factors results in less replication initiation in S phase. This forces each fork to travel further to complete genome duplication and is thought to increase the probability of fork stalling and cells entering mitosis without a fully duplicated genome [23]. Fewer licensed origins also means a reduction in dormant origins that are able to rescue stalled replication forks [8]. A number of firing factors are limiting for replication initiation and so increases in protein levels, as is often seen in cancer, can result in increased replication initiation and can disrupt the temporal pattern of origin firing [53]. Activation of the oncogenes Cyclin E or c-Myc can also cause increased and deregulated replication initiation [47,54]. In addition to increasing replication and transcription collisions, as discussed above, increased replication initiation may deplete essential replication factors, such as dNTPs [50], both of these cause replication stress.

Re-replication can also occur if regulatory mechanisms fail and allow licensing of replicated DNA in S phase, as is seen following overexpression of Cdt1 or Cdc6 [55]. Re-replication results in gene amplifications and genome instability [56]. If re-replication is infrequent it can cause replication stress by increasing the probability of fork stalling due to the large distance between re-replication origins and a lack of converging forks to rescue replication. Re-replication may also cause replication stress by depleting replication components and increasing collisions between replication and transcription.

Activation of oncogenes or inactivation of tumour suppressors often deregulates the CDK-pRB-E2F pathway [57], therefore driving unscheduled S phase entry. This uncontrolled proliferation is thought to induce replication stress through many of the mechanisms discussed, including deregulation of replication origin licensing and firing, exhaustion of replicative factors and increasing replication and transcription collisions [23,47,50,55,58].

## 3. DNA Replication Stress Checkpoint Response

**Figure 1:**
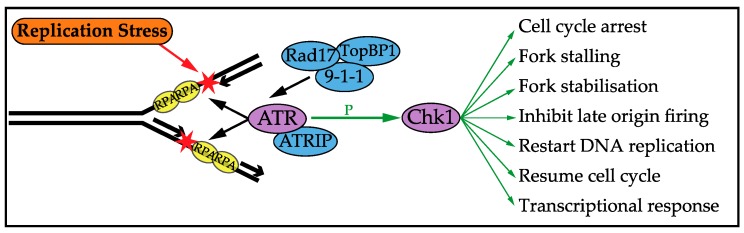
DNA replication stress is the slowing down or stalling of replication forks, which exposes single-stranded DNA (ssDNA). ssDNA is bound by Replication Protein A (RPA), which recruits proteins to the stalled fork (recruitment shown with black arrows). This activates the sensor kinase Ataxia Telangiectasia and Rad3-related protein (ATR), which phosphorylates and activates the effector kinase Chk1. Chk1 phosphorylates a wide range of targets in the cell to carry out the DNA replication stress checkpoint functions shown.

In order to tolerate DNA replication stress, the cell has evolved a checkpoint response, conserved from yeast to man, which prevents DNA damage and genome instability [59]. The checkpoint response is triggered by the extended lengths of ssDNA exposed during replication stress, likely due to continued helicase action once the polymerase has stalled [60]. The cellular response to DNA replication stress has been extensively reviewed previously [7,19,20,59,61,62,63,64,65]. ssDNA is bound by a ssDNA binding protein, which protects vulnerable ssDNA and recruits the checkpoint sensor kinase; in mammalian cells these proteins are Replication Protein A (RPA) and ATR (Ataxia Telangiectasia and Rad3-related protein), respectively [14,66,67]. ATRIP (ATR Interacting Protein) is recruited with ATR [68]. Rad17 is also recruited, which loads the 9-1-1 complex, which recruits TopBP1 to fully activate ATR [63,69,70]. ATR is a serine/threonine kinase of the PI-3-like kinase family that phosphorylates, among other targets, the checkpoint effector kinase Chk1 [71,72], as summarised in Figure 1. Replication stress primarily induces this ATR-Chk1 pathway, whilst the response to DNA DSBs mainly depends on ATM-Chk2 signalling. However, crosstalk between the two pathways is seen and ATR and Chk1 can have distinct and independent roles in the DNA replication stress checkpoint response [73,74,75]. Once activated, Chk1 phosphorylates a wide range of targets, altering their level and activity, thereby activating the checkpoint functions discussed below.

### 3.1. Cell Cycle Arrest

To ensure DNA replication is completed before a cell enters mitosis, the replication stress checkpoint arrests the cell cycle by inhibiting CDK activity. CDK activity is constrained by Wee1-dependent phosphorylation [76], which is removed by the Cdc25 phosphatase. Chk1 acts to increase Wee1 activity and targets Cdc25 for Ubiquitin-dependent degradation and therefore increases CDK inhibitory phosphorylation, thereby arresting the cell cycle [63,77].

### 3.2. Stalling and Stabilising Replication Forks

Under conditions of replication stress in which obstructions or depletion of key components may stall replication forks, other ongoing replication forks must be stalled in a checkpoint-dependent manner to prevent further replication stress or DNA damage [20,78]. Allowing replication to continue could further deplete replication components or expose such high amounts of ssDNA that RPA cannot protect all vulnerable ssDNA and replication catastrophe results [66]. The checkpoint can also upregulate RNR activity to increase the levels and prevent exhaustion of dNTPs [62]. To prevent the dissociation of the replisome and the formation of aberrant DNA intermediates, stalled forks must be stabilised in a process dependent on Chk1 [62,79,80,81]. In order to stabilise stalled replication forks, a fork protection complex is formed containing factors such as Rad51, Fanconi Anemia Complementation Group D2 (FANCD2) [82], PCNA [31], Cdc7 [83,84], Timeless and Tipin [78]. Formation of this complex is thought to be essential for stalling and stabilising replication forks.

### 3.3. Control of Origin Firing

The DNA replication stress checkpoint also regulates the firing of replication origins. ATR and Chk1 prevent new replication factories from forming and therefore inhibit late origin firing, directing replication components to sections of the genome already undergoing replication [22]. In contrast to the global inhibition of origin firing, dormant origins local to stress are fired to complete replication. This is thought to be stochastic firing of dormant origins, which due to fork stalling have not been passively replicated and disassembled [10,22]. Together these mechanisms ensure that replication is completed in regions experiencing stress, but no further forks are put at risk of stalling in unreplicated regions.

### 3.4. Replication Restart

Following the resolution of replication stress, DNA replication must be completed through a number of different replication restart mechanisms, discussed in detail in [21,85]. Following short periods of stress, a stalled fork may be restarted via remodelling by helicases. Replication can also be restarted directly from an intact but stalled replication fork in a process dependent on Rad51 and X-Ray Repair Cross Complementing 3 (XRCC3), but not involving Homologous Recombination [79]. This process is thought to involve Rad51 coating ssDNA at stalled forks and mediating strand invasion, which allows replication restart. Forks collapse after longer periods of stress and can be processed into fork-associated DSBs [86]. This then requires repair mechanisms such as Homologous Recombination and the local new firing of dormant origins to complete DNA replication.

## 4. The Transcriptional Response to DNA Replication Stress

**Figure 2:**
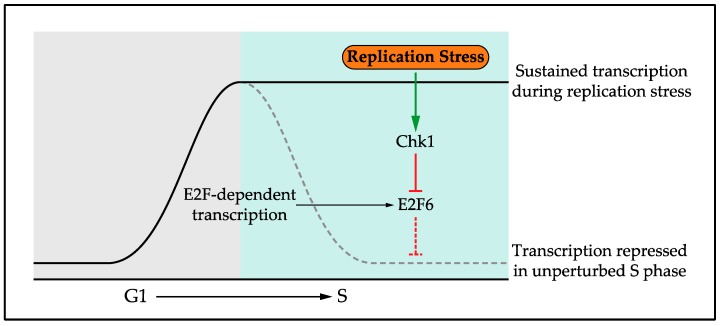
In the response to DNA replication stress the checkpoint effector kinase inactivates a repressor, resulting in sustained G1/S cell cycle transcription. This transcriptional response has a key role in the tolerance to DNA replication stress. This response is conserved from yeast to man, with the mammalian names shown here.

The role of post-translational modifications in regulating and coordinating the response to DNA replication stress has been widely studied [87,88]. Phosphorylation is a key element of the checkpoint response through ATR and Chk1 kinase activity, but ubiquitination and sumolation are also important [89]. However, until recently the role of transcription in the Replication Stress Response (RSR) was largely unknown. Initially the response to replication stress, including the transcriptional response, was considered together with the response to DNA damage and collectively named the DNA Damage Response (DDR). However, it has become increasingly clear that these represent independent responses, in signalling, function and outcome, prompting the authors in a recent review to use the subheading “The RSR: time to fly *solo* from the DDR” [90]. In line with this, work carried out in the fission yeast *Schizosaccharomyces pombe* established a transcriptional response that is specific to replication stress. This work showed that G1/S cell-cycle-regulated transcription is maintained in response to replication stress [91,92,93,94,95,96]. Interestingly, this is a specific function in the response to replication stress as G1/S transcription is instead inactivated in response to DNA damage, in a checkpoint-dependent manner [97,98]. Subsequent work in the budding yeast *Saccharomyces cerevisiae* [99,100] and human cells [101] established that this transcriptional response to replication stress is conserved from yeast to man [61]. G1/S transcription is a wave of transcription encoding many components required in S phase, such as those required for DNA replication and repair. Activation of G1/S transcription in G1 phase drives cell cycle entry and transcription is subsequently repressed upon S phase entry. G1/S transcription encodes its own repressor, setting up a negative feedback loop to turn off transcription [101,102]. In response to DNA replication stress, G1/S cell cycle transcription is maintained through the checkpoint-dependent phosphorylation and inhibition of this repressor, Nrm1 in yeast and E2F6 in mammalian cells [91,94,99,100,101], Figure 2.

### 4.1. Role of the Replication Stress Transcriptional Response

The conservation of this transcriptional response and its regulatory mechanism suggests an important role in the cellular response to DNA replication stress. However, in budding yeast active protein synthesis is not required for cell viability following replication stress [103], suggesting a non-essential role for the transcriptional response. In contrast, in human cells maintaining G1/S transcription is a key element of the checkpoint response [101,104]. In mammalian cells G1/S cell cycle transcription is controlled by the E2F family of transcription factors. E2F-dependent transcription during G1 depends on the E2F1-3 transcriptional activators, whereas inactivation during S phase depends on the E2F target and transcriptional repressor E2F6 [105,106,107]. In response to replication stress the checkpoint protein kinase Chk1 maintains transcription via phosphorylation and inactivation of E2F6 [101]. This transcriptional response is required in mammalian cells for an efficient DNA replication stress checkpoint to prevent DNA damage and genome instability [104].

Stress responses generally induce the transcription of a separate gene network [108]. The transcriptional response to replication stress, where an ongoing transcriptional network is maintained, is therefore atypical. Key DNA replication control proteins and checkpoint effector proteins are E2F targets and are therefore expressed during the G1 to S transition. Recent work in mammalian cells reveals that many of these proteins have short half-lives; therefore, during a replication stress checkpoint cell cycle arrest, sustained E2F-dependent transcription is required to maintain the levels of these proteins [104]. In some cases, E2F-dependent transcription is also required for up-regulation of checkpoint effector proteins. Sustained E2F-dependent transcription and the resulting maintenance of protein levels is required for key checkpoint functions, including the stalling and stabilisation of replication forks, the formation of the protective fork complex and the resolution of stalled forks once the stress has been relieved. However, this transcriptional response is not seen to have a role in arresting the cell cycle. Importantly, sustained E2F-dependent transcription is sufficient to form a protective fork complex, allow the restart of DNA replication following stress and prevent DNA damage in checkpoint-compromised conditions [104]. The transcriptional response to DNA replication stress is therefore required and sufficient for key functions of the checkpoint response to prevent DNA damage and allow cell viability. The number of E2F targets needed to be maintained for an efficient checkpoint response remains unknown. Specific E2F targets, such as Chk1 and RRM2, have important roles in the checkpoint response and have been proposed as “replication stress buffers” [24]. Up-regulation of these proteins is protective in checkpoint-compromised and oncogenic mouse models [109,110]. Whilst it is unlikely that sustaining the expression of one specific E2F target alone is sufficient to prevent replication stress-induced DNA damage, the actual number of E2F targets involved remains unknown.

## 5. Regulation of the Transcriptional Response

Sustained E2F-dependent transcription has an essential role in the DNA replication stress checkpoint response. However, this transcriptional response must be tightly regulated to prevent damaging effects. Inappropriate expression of individual E2F targets, including Cyclin E, Cdc6 and Cdt1, causes DNA replication stress and genome instability [47,55,111]. In addition, maintaining E2F-dependent transcription during S phase would result in increased transcription of many targets, which is likely to increase the chance of collisions between replication forks and transcriptional bubbles.

### 5.1. Confining the Transcriptional Response to Replication Stress

**Figure 3:**
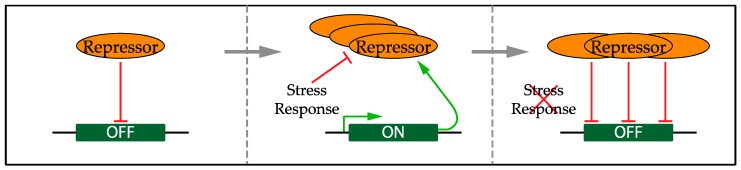
Schematic showing a general network wiring consisting of a stress response inhibiting a negative feedback loop and a repressor with the capacity to repress its own transcription. This would allow for rapid down-regulation of transcription and fast changes in the proteome during the recovery from the stress response.

The transcriptional response to DNA replication stress involves the inactivation of a negative feedback loop. Interestingly, this molecular mechanism is used in several transcriptional responses to genotoxic stress. In budding yeast, DNA replication stress also results in Dun1-dependent inactivation of a negative feedback loop involving the repressor Crt1 [112]. This primarily induces RNR genes involved in tolerance to DNA replication stress; however, this transcriptional response is less well-studied in mammalian cells. The mammalian homologue Rfx1 is also regulated by a negative feedback loop with DNA replication stress inactivating Rfx1, resulting in Rfx1 and RRM2 up-regulation [113], however the importance of this for replication stress tolerance is not known. Although increased RRM2 levels are protective in ATR mutant mice [110], work has indicated that UV-irradiated mammalian cells do not strongly increase dNTP levels [114]. Regulation of a negative feedback loop is also seen in the SOS response in *Escherichia coli* involving the repressor LexA [115]. During recovery from the DNA damage checkpoint response, regulation of transcription is also mediated via a negative feedback loop, involving Mdm2 and p53 [116,117]. This network wiring, with the repressors having the capacity to repress their own expression, would ensure the fast inactivation of transcription during recovery from the genotoxic stress, Figure 3.

The conservation of this mechanism suggests that these DNA damage and replication stress-induced transcriptional responses may be deleterious once the problems have been resolved. Although the exact defects remain to be established, data suggests that persistent expression of G1/S targets during S phase results in genome instability in both yeast and mammalian cells ([102,118] and unpublished data). This suggests an important role for the repression of G1/S transcription outside of the G1 to S phase transition and, perhaps, after recovery from DNA replication stress. In mammalian cells, there is evidence of checkpoint-dependent degradation of checkpoint effector proteins such as Chk1 [119,120], but how widely this mechanism is used by the cell remains to be determined. This additional level of regulation would further ensure that checkpoint-dependent gene expression is turned off once the checkpoint has been satisfied. The combination of this transcriptional network inactivation, short half-lives and checkpoint-dependent degradation would mean rapid changes in the proteome to inactivate the checkpoint response. Future research will reveal whether rapid down-regulation of DNA structure checkpoint-dependent gene expression is generally important for the maintenance of genome stability.

### 5.2. DNA Replication Restart

Following checkpoint inactivation, DNA replication must be restarted; the mechanism signalling this has not been fully established [85,121]. The βTrCP-dependent degradation of Claspin is required for efficient termination of Chk1-dependent checkpoint signalling and subsequent recovery of cell cycle progression [122,123]. Phosphatase activity can also reverse checkpoint signalling and this is a key mechanism required for replication fork restart [124,125]. One could speculate that the particular transcriptional response to replication stress, where gene expression is maintained, could have an important contribution to checkpoint recovery. A combination of sustained transcription and proteins with short half-lives would result in high turnover rates. Therefore, proteins post-translationally modified by the checkpoint would be replaced by new and unmodified proteins as soon as the checkpoint is satisfied. This could act as a robust inactivation of checkpoint signalling in order to allow and signal for DNA replication restart. Enzymes, such as phosphatases, have some role in this checkpoint inactivation and recovery [124,125]. However, a mechanism relying on turnover rates may have a number of advantages. It would be a widespread mechanism to quickly replace post-translationally modified proteins without the need for individual enzymes to remove each type of post-translational modification and could therefore be a faster and more robust way of removing checkpoint-dependent modifications. Checkpoint inactivation dependent on inherent degradation could prevent indefinite checkpoint activity that would be detrimental for the cell. In addition, as discussed above, the response containing a repressor poised to function as soon as checkpoint signalling is inactivated would ensure the fast inactivation of further checkpoint signalling. Importantly, this suggested mechanism would also directly link checkpoint inactivation and DNA replication restart.

## 6. The Replication Stress Transcriptional Response and Oncogenic Activity

**Figure 4:**
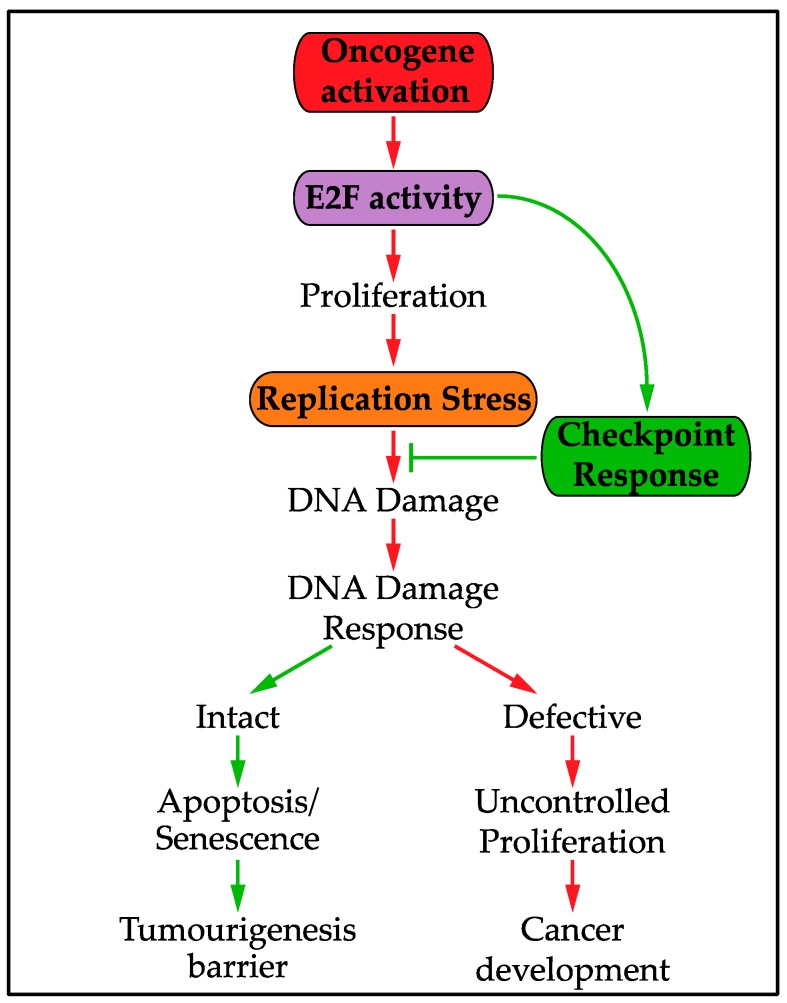
Many oncogenes deregulate E2F activity, thereby driving S phase entry and uncontrolled proliferation, resulting in oncogene-induced replication stress and DNA damage. The DNA damage response acts as an initial barrier to tumourigenesis, but replication stress causes genome instability driving mutations that bypass the DNA damage checkpoint. However, E2F activity is also required for tolerance to oncogene-induced replication stress to prevent DNA damage.

Maintenance of E2F-dependent transcription in response to DNA replication stress is important to prevent replication stress-induced DNA damage. However, increased E2F activity is thought to be a driving force in causing oncogene-induced replication stress. E2F-dependent transcription is deregulated following activation of many oncogenes, such as Myc, Ras and Cyclin/CDKs, or inactivation of some tumour suppressors, such as CDK Inhibitors and pRb, which all regulate the signalling pathway upstream of E2F [57,106,107,126]. This deregulation of E2F-dependent transcription, which controls the G1 to S phase transition, drives unscheduled S phase entry and uncontrolled proliferation [51]. As discussed previously, this uncontrolled proliferation is thought to result in oncogene-induced replication stress through a number of possible mechanisms. In the context of oncogene-induced replication stress, E2F-dependent transcription is required to both drive and tolerate replication stress [104]. This dual role creates a likely increased dependence on E2F activity in cancer cells, Figure 4. Cancer cells experiencing high levels of replication stress are expected to require much higher levels of E2F activity and checkpoint function compared to normal cells. This mechanism of tolerance could be exploited in future cancer treatments to target cancer cells without harming healthy cells.

## 7. Future Perspectives

The identification of a transcriptional response to DNA replication stress [91,92,93,94,95,96,99,100,101] and understanding its key role in the checkpoint response [104] opens up possible new areas of research [127]. Understanding the complex interactions between transcription, translation, post-translational modifications and degradation rates, which together control the activity of checkpoint effector proteins, would enhance our knowledge of the DNA replication stress checkpoint. This integrated network wiring could allow the cell to quickly re-adjust the proteome once the stress has been resolved. This may be relevant to other signalling pathways, in particular, other stress responses.

It will be important to establish whether the tolerance to replication stress is dependent on a few key targets, or the up-regulation of the whole G1/S transcriptional network. This could guide the best approach to potentially exploit this tolerance mechanism in cancer treatment. Simultaneously targeting the transcriptional tolerance mechanism to replication stress and DNA repair mechanisms may be very effective to prevent continued proliferation of oncogenic cells. Overall, transcription has only recently been identified to have a key role in tolerating DNA replication stress, which provides interesting new avenues of research to fully understand and exploit the DNA replication stress checkpoint.
